# Conjunctival melanoma with pronounced central corneal invasion: One-year relapse free follow-up

**DOI:** 10.1016/j.ajoc.2024.102162

**Published:** 2024-08-30

**Authors:** Colya N. Englisch, Tim Berger, Fidelis Flockerzi, Max Bofferding, Berthold Seitz

**Affiliations:** a Department of Ophthalmology, Saarland University, 66421 Homburg, Saar, Germany; b Department of Experimental Ophthalmology, Saarland University, 66421 Homburg, Saar, Germany; c Institute of Pathology, Saarland University, 66421 Homburg, Saar, Germany

**Keywords:** Conjunctival melanoma, Conjunctival melanocytic intraepithelial lesion, Primary acquired melanosis, Corneal involvement, Surgical removal, Mitomycin c

## Abstract

***Purpose*:**

Conjunctival melanoma with large corneal involvement is a rarity. We here present a case of conjunctival melanoma with pronounced central corneal involvement.

***Observation*:**

A 69-year-old fair white male presented with a visual axis impeding corneal nodular lesion with associated conjunctival melanosis. Tumor excision with intraoperative mitomycin c (0.02 %) application for 180 seconds and amniotic membrane transplantation for defect coverage was performed in retrobulbar anesthesia. Histopathological evaluation revealed the nodular lesion to be a conjunctival melanoma (pT1a) with associated conjunctival melanocytic intraepithelial lesion (C-MIL).

***Conclusion and importance*:**

Most conjunctival melanomas with corneal affection reach a radial corneal involvement of 1 mm. The here reported case accounted for 4 mm, which is seldom and therefore an important report. Surgical excision followed by intraoperative and postoperative mitomycin c exposure was a successful primary treatment. Currently there are no signs of tumor relapse in any part of the eye or the organism 12 months after excision. However, the long-term follow-up needs to be awaited.

## 1 Introduction

Melanoma is the most frequent malignant ocular lesion. Here the most common localization corresponds to the uvea or more specifically the choroid.^1^^,^^2^ Conjunctival melanoma (CM) is in contrast quite a rare entity only found in 5–7% of ocular cases of melanoma.^3^ CM can arise *de novo* or develop from pre-existing lesions including conjunctival nevus and most frequently from conjunctival melanocytic intraepithelial lesion (C-MIL)^4^ – before known as primary acquired melanosis (PAM).^2^^,^^5^^,^^6^ Minimal corneal involvement is often observed in conjunctival melanoma, but rarely reaching the optical axis.^7^ This case report presents an impressive case of diagnosed CM with pronounced corneal involvement and one-year relapse free follow-up after surgical excision.

## 2 Case presentation

A 69-year-old fair-white Caucasian male patient with blue iris color, Fuchs endothelial dystrophy, and incipient corticonuclear cataract on both eyes presented with symptoms of pruritus, blurred and decreased vision for ten days on the left eye. No previous surgeries, or systemic treatments including chemotherapy, corticosteroids, or ocular radiation were known. Medical history was uneventful except arterial hypertension. There were no hints for present or former cutaneous melanoma, dysplastic nevus syndrome, uveal melanoma, neurofibromatosis, or acquired immunodeficiency syndrome. The patient didn't report any form of conjunctival pigmentation before onset of symptoms. Family history was evenly empty with respect to conjunctival, uveal, or cutaneous melanoma, dysplastic nevus syndrome, and neurofibromatosis.

**Fig. 1:**
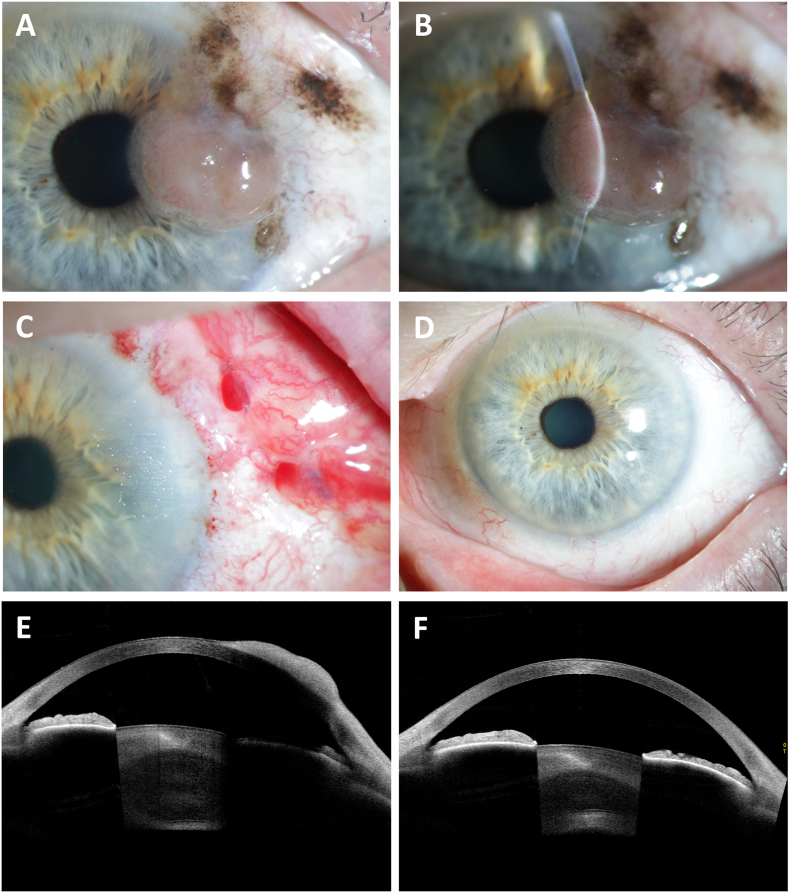
Preoperative slit lamp photo documentation demonstrating a visual axis impeding corneal nodular lesion with associated conjunctival melanosis (A and B). Postoperative findings after one day (C), and one year (D). Anterior segment OCT showing a prominent, hyperreflective lesion preoperatively (E) and one year after complete tumor excision (F).

Slit lamp examination displayed a lateral nodular corneal lesion from 2 to 4 o'clock with an associated lateral C-MIL from 1 to 4 o'clock on the left eye (Fig. 1A and B). The radial corneal involvement accounted for 4 mm and ultimately reached the central corneal portions. The tumorous lesion demonstrated a pale pink color with a base measuring 4 × 2 mm and a thickness of 720 μm. Corneal involvement appeared to be mostly restricted to the epithelium, as supported by anterior segment optical coherence tomography (OCT),^8^ although stromal infiltration could not be ruled out, since Bowman's layer couldn't be assessed in total (Fig. 1E). Both eyelids appeared free of lentigo maligna. Best-corrected decimal visual acuity (BCVA) was 0.5, which was lower than that of the neighbor right eye with 1.0. In addition, secondary astigmatism (−6.25 diopters à 171°) and hyperopia (+7.5 diopters) were increased compared to the non-affected eye (−0.25 diopters à 37°, +3.0 diopters, respectively). Based on the clinical appearance and development, the diagnosis of CM with corneal invasion reaching the optical axis was highly suspected.

Clinical and sonographic examination of preauricular, submandibular, and cervical lymph nodes by the otolaryngologist remained without pathological finding. Fundoscopy and posterior segment OCT, chest-X-ray, cranial magnetic resonance imaging, abdominal sonography and computed tomography were labeled as age-appropriate with no evidence for metastases.

**Fig. 2:**
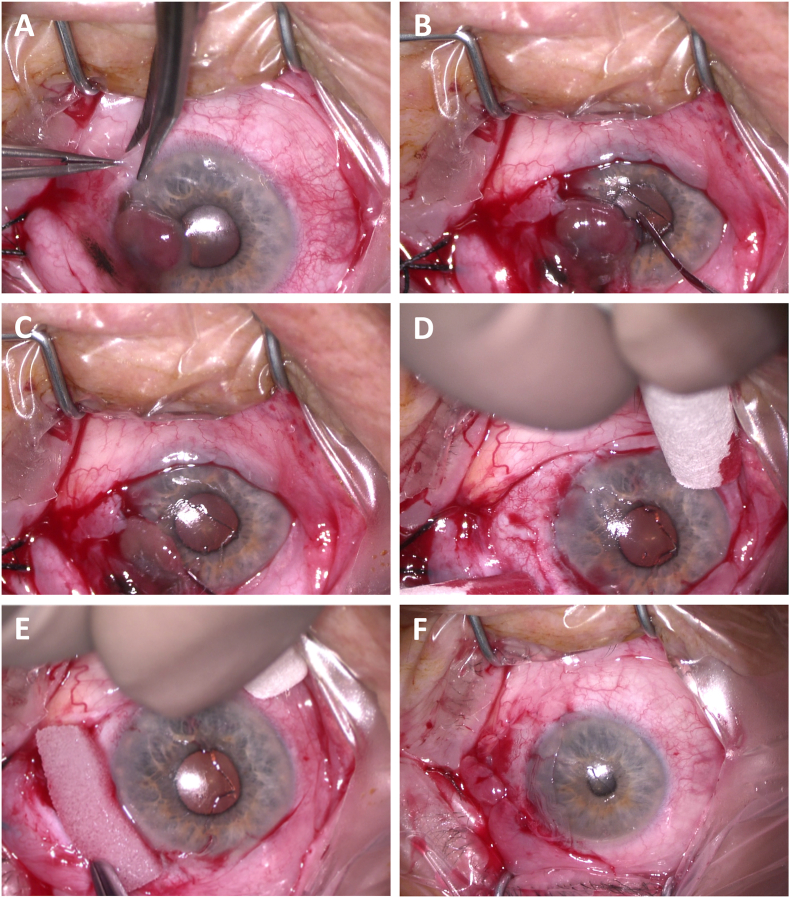
Intraoperative photo documentation. The conjunctival part of the tumor was excised with a Wescott scissor (A), while the corneal part of the conjunctival melanoma was removed with a hockey knife (B, C). After complete removal of the corneal portion, no defect of Bowman's layer was visible (D). Following surgical excision, topical mitomycin c (0.02 %) was applied in a soaked microsponge for 180 seconds (E). The defect was covered with an amniotic membrane transplantation (graft, F).

Three weeks after initial presentation, tumor excision (Fig. 2) was performed under retrobulbar anesthesia. The conjunctival part of the tumor was removed with a safety margin of 4 mm, while the corneal part of the tumor could be removed in one piece using a hockey knife without a visible defect of Bowman's layer. After tumor removal, topical mitomycin c (0.02 %, MMC) soaked in a microsponge was applied to the bare sclera at the excision site for 3 min and washed away using balanced salt solution. Amniotic membrane transplantation (as episcleral graft) was performed to cover the conjunctival defect.

**Fig. 3:**
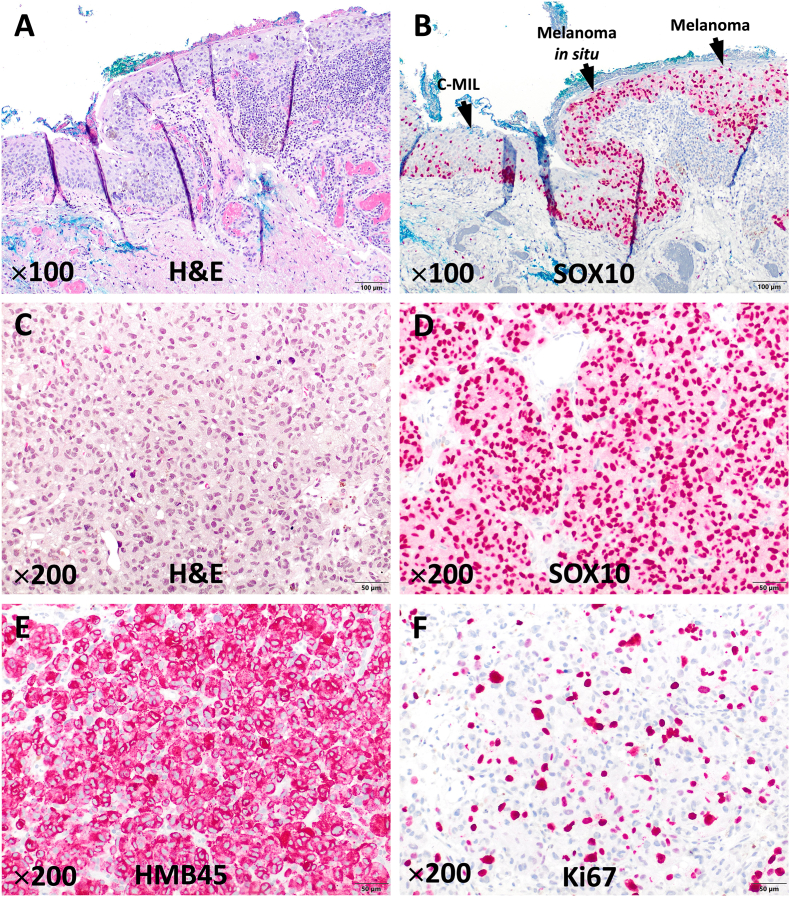
Histopathological analysis. A–B: Overview: Conjunctival melanocytic intraepithelial lesion (C-MIL)/invasive melanoma: On the left side, only few melanocytic cells (SOX10-positive) scattered in the basal layer. Towards right side, increased number of melanocytic cells with atypia and vertical spread (C-MIL). On the right side (A and B) invasive melanoma with invasion of the subepithelial connective tissue. (A: Hematoxylin & Eosin staining, B: SOX10 staining; 100 × magnification). C–F: High magnification: Corneal subepithelial connective tissue with invasion of partially pigmented tumor cells with atypia (C), positivity for SOX10 (D) and HMB45 (E), as well as increased proliferation activity (F) (C: Hematoxylin & Eosin staining, D: SOX10 staining, E HMB45 staining, F: Ki67 staining; 200 × magnification).

Histopathological analysis revealed a C-MIL with severe atypia (melanoma *in situ*) with focal transition towards invasive melanoma. In both corneal and conjunctival samples, atypical melanocytic cells with horizontal and suprabasilar extension, as well as arrangement in intraepithelial confluent nests were detected (C-MIL high grade). At least focally, nests of tumor cells with nuclear atypia, mitotic figures, increased proliferation activity (Ki67 index up to 10 and 15 %, respectively) as well as positive staining for SOX10 and HMB45 with stromal invasion were detected; thickness of invasive melanoma was less than 2 mm (Fig. 3). According to 8th edition of TNM classification, tumor stage of invasive melanoma component was pT1a. In the corneal sample, invasive melanoma focally involved the margin of the sample, which is why complete surgical resection could not be achieved based on histopathological analysis.

A topical treatment with ofloxacin (3 mg/ml) eye drops three times daily for three days followed. Then, three days postoperatively, the first of three planned MMC cycles was started with 0.02 % MMC eye drops four times daily, ofloxacin eye drops four times daily (3 mg/ml), dexamethasone eye drops four time daily (1.315 mg/ml), and dexpanthenol eye drops eight times daily (50 mg/g). The cycle lasted seven days and was repeated twice in one-week intervals. In the interim time, the same treatment combination was administered but without MMC. The postoperative clinical appearance was proper and improved quickly. Fig. 1C displays the clinical appearance one day postoperatively. The clinical appearance showed in Fig. 1D was already reached one month postoperatively and remained stable until one year postoperatively. Fig. 1F shows the one-year anterior segment OCT scan which is also free of pathological findings. In addition, BCVA rapidly improved to 1.0 at day ten postoperatively. Astigmatism and hyperopia also improved to –0.25 diopters à 90° and +3.0 diopters, respectively. Accordingly, there were neither signs of lasting corneal scarring nor vision deterioration at that time point nor during the follow-up visits. The patient was controlled every month in the first postoperative half year, and then after six months. After 12 months, the patient remained lesion free in both eye and body.

Written informed consent was obtained from the patient and the study was conducted in accordance with the tenets of the Declaration of Helsinki.

## 3 Discussion and conclusions

CM is a rare malignancy with poor prognosis, arising from melanocytes within the basal cell layer of the eye surface-lining conjunctival epithelium.^3^^,^^5^^,^^9^, ^10^, ^11^, ^12^ Most ophthalmologists will treat no more than one case of CM in their entire clinical practice.^13^ However, in contrast to uveal melanoma, incidence of CM has increased in Northern countries, as reported for Sweden and the United States along the past decades.^2^^,^^3^^,^^14^^,^^15^ Etiologically, CM can originate from conjunctival nevi, C-MIL, or *de novo*, but origin is not a prognostic relevant factor for clinical outcome.^6^ Most CM arise from C-MIL, and inversely C-MIL is a significant risk factor for development of invasive melanoma.^6^^,^^16^^,^^17^ In this context, C-MIL with severe atypia also described as melanoma *in situ* is the most important entity.^16^^,^^17^ CM has a wide spectrum of clinical manifestations but usually appears as pigmented or tan elevated lesion.^12^ Frequent findings include described spots and/or lumps, irritation, or pain.^7^^,^^18^ The presence of both prominent feeder vessels and proximate flat C-MIL is also common,^7^ whereas in the presented case the former were absent (Fig. 1). Localizations of the CM include the bulbar conjunctiva, the fornix, tarsus, plica semilunaris, and caruncle.^7^^,^^12^ CM are localized in approximatively 90 % of cases on the bulbar conjunctiva.^7^^,^^12^ Here, tumor localization was mostly described in the lateral eye quadrant with 63 %.^7^ In a study from Shields et al. involving 150 CM patients, mean proximity of the tumor to the corneal limbus amounted 2 mm.^7^ The malignancy reached the limbus in 61 % of cases. Interestingly, the mean radial tumor penetration of the cases with corneal affection was no more than 1 mm,^7^ which is in clear contrast to the presented case. In these tumors with corneal affection, intrastromal invasion was only observed in one single case.^7^ Involvement of the cornea is therefore not a rare phenomenon; however, it is seldom as pronounced as in the here presented case.

Important differential diagnoses to CM are different conjunctival pigmentary abnormalities such as PAM, complexion-associated melanosis, secondary acquired melanosis, melanocytic hyperplasia, standard melanocytic nevus, or blue nevus as indicated by Shields et al.^12^ As a matter of fact, the here presented corneal involvement could also correspond to a primary corneal melanoma. However, primary corneal melanoma is extremely rare.^19^ During the last 130 years only a few case reports (e.g.,^20^, ^21^, ^22^, ^23^) of primary corneal melanoma have been published as indicated by Panagiotou and colleagues.^19^ In most cases where literature is available, however, there was no described conjunctival pigmentation. Although the corneal mass alone in our case would be compatible with the seldom described primary corneal melanoma, the presence of the C-MIL rather speaks against. Indeed, malignant cells were observed in both conjunctival and corneal samples suggesting the melanoma source being of conjunctival nature with tumor extension towards the cornea (C-MIL with transition to invasive melanoma). With respect to the pronounced corneal involvement the designation of secondary corneal melanoma would seem to be accurate.

The case we presented here showed a two-week long history of symptoms. As described above, transition of precursor (melanoma *in situ*) to invasive cancer (melanoma) was shown in histopathological analysis, evidencing the complex challenge of combining entire tumor excision with optimal visual outcome and relapse free follow-up. As suggested, surgical excision is often a good choice for localized and early CM manifestations.^24^ While the no-touch technique of microsurgical excisional biopsy with 3–4 mm tumor free margins belongs to the most preferred for CM,^25^ corneal involvement can become challenging. In this context, alcohol corneal epitheliectomy has been recommended.^25^ The putative C-MIL was excised as recommended by Shields and colleagues^25^ – a leading group in conjunctival melanoma research^26^ – since associated C-MIL can enhance further melanoma development.^16^^,^^17^ The clinical result was good and not concomitant with corneal scaring or vision deterioration, which is quite noteworthy, since the corneal lesion had a radial diameter of 4 mm directly impeding the optical axis. However, histopathological analysis didn't demonstrate entire tumor resection. For such cases adjunctive MMC treatment is a known option, that can be used intra- and postoperatively, in cases of both *in toto* and incomplete excision.^27^, ^28^, ^29^, ^30^ Its safe application in corneal neoplasia affecting the visual axis is documented.^31^ Other described adjuvant options include cryo- or plaque radiotherapy, and external irradiation.^7^^,^^27^ In this context of neoadjuvant or adjuvant treatment the use of targeted therapies is increasingly investigated in CM, especially in advanced stages of disease.^32^, ^33^, ^34^ In the presented CM case, surface reconstruction was reached through amniotic membrane graft transplantation, as commonly done for bulbar tumors,^35^ although other options exist.

Nevertheless, CM is known to regularly relapse. Anastassiou et al. described CM recurrence in approximatively 49.3 % of patients with incomplete surgical removal, deep tumor invasion that means deeper than substantia propria, amelanotic and mixed pigmentation being indicators of recurrence.^27^ Similar numbers were found elsewhere.^36^ A previous study also suggested that melanoma not touching the limbus was a risk factor for recurrence and metastasis.^7^ No studies are known by the authors where the effect of severe corneal involvement on metastasis and prognosis was addressed. Mortality is associated with incomplete surgical removal, deep tumor invasion including in the orbit, nodular or mixed growth patterns, and tumor location in the caruncle, palpebral, plical, and forniceal conjunctiva.^7^^,^^27^^,^^36^^,^^37^ Tumor growth can indeed range from localized pattern to the invasion of neighbored structures including the orbit, the nasolacrimal system, nasal cavity and paranasal sinuses.^38^ In the context of localized intraocular extension, the Bowman's layer plays an important role as tumor cell barrier.^29^^,^^38^ With respect to filiation, both lymphatic and hematic ways are described.^11^^,^^38^ After five years, metastasis will be detected in about 15 % of patients.^7^^,^^38^ Common metastasis sites include ipsilateral preauricular and submandibular lymph nodes, the brain, lung, and liver.^7^^,^^39^ For these reasons, patients should be checked regularly in four-to-six-month intervals for metastasis, including ophthalmological, otolaryngological, and general physical examination. Chest X-rays and cranial MRI should be done yearly.

In conclusion, the presented case is important due to the pronounced central corneal CM involvement, which reached a size only rarely described until now. However, follow-up is needed to enable a statement concerning recurrence in this case.

## Declarations and patient consent

The study adhered to the tenets of the Declaration of Helsinki. Anonymous case reports are waived by the Ethics Committee of the Saarland Medical association. The patient formally assigned the written consent for publication.

## Funding

No funding or grant support.

## Authorship

All authors attest that they meet the current ICMJE criteria for Authorship.

## CRediT authorship contribution statement

**Colya N. Englisch:** Writing – original draft, Visualization, Data curation. **Tim Berger:** Writing – review & editing, Validation, Supervision, Data curation. **Fidelis Flockerzi:** Writing – review & editing, Investigation. **Max Bofferding:** Investigation. **Berthold Seitz:** Writing – review & editing, Resources.

## Declaration of competing interest

None. The authors certify that they have no affiliations with involvement in any organization or entity with any financial interest in the subject matter or materials discussed in this manuscript.

## List of abbreviations

BCVA: Best corrected decimal visual acuity
CM: Conjunctival melanoma
C-MIL: Conjunctival melanocytic intraepithelial lesion
MMC: Mitomycin C
OCT: Optical coherence tomography
PAM: Primary acquired melanosis
